# On Quality Analysis of Filtration Methods for Bathymetric Data in Harbour Areas through QPS Qimera Software

**DOI:** 10.3390/s23115076

**Published:** 2023-05-25

**Authors:** Witold Kazimierski, Małgorzata Jaremba

**Affiliations:** 1 Faculty of Navigation, Maritime University of Szczecin, Waly Chrobrego 1–2, 70-500 Szczecin, Poland; 2 Independent Researcher, 70-500 Szczecin, Poland

**Keywords:** bathymetry, multibeam echosounder, digital bottom model, data filtration, hydrography, surface modeling

## Abstract

This paper presents an assessment of the quality of selected filtration methods for the postprocessing of multibeam echosounder data. In this regard, the methodology used in the quality assessment of these data is an important factor. One of the most important final products derived from bathymetric data is the digital bottom model (DBM). Therefore, quality assessment is often based on factors related to it. In this paper, we propose some quantitative and qualitative factors to perform these assessments, and we analyze a few selected filtration methods as examples. This research makes use of real data gathered in real environments, preprocessed with typical hydrographic flow. The methods presented in this paper may be used in empirical solutions, and the filtration analysis may be useful for hydrographers choosing a filtration method for DBM interpolation. The results showed that both data-oriented and surface-oriented methods can be used in data filtration and that various evaluation methods show different perspectives on data filtration quality assessment.

## 1. Introduction

Bathymetry, understood as seabed topography, is one of the key attributes of the underwater environment, and it has been used by humans since the beginning of exploration of water environments. Traditionally, it has been used for onboard and onshore maritime information systems [1]; however, nowadays more and more usages exist. For example, in [2] the focus is on dredging operations, and in [3] an analysis of seabed changes is presented. General environmental modelling is presented in [4], and in [5] multiple applications related principally to the safety of navigation for all sea users and digital terrain modeling are explored. Further examples include the comparative navigation approach [6], in which bathymetry plays a crucial role as a reference, and bathymetric measurement methodologies are used to model water-column information, for example, in [7], where the abundance of giant kelp in the water column was detected with echosounder backscatter.

The use of multibeam echosounders (MBESs) has become a standard approach in seabed modelling, especially large-area surveys. They allow 100% coverage in a survey, which is a requirement for the highest orders of surveys according to the International Hydrographic Organization (IHO) standard [8]. MBES systems were developed and used first in the 1980s, and since then have gained huge popularity; in the scientific field, they have become a main target of interest. They did not, however, replace single-beam echosounders (SBESs), the use of which is still considered reasonable for many applications. For example, in [9], a method combining a low-cost SBES with a dual-frequency differential high-precision global navigation satellite system (GNSS) is presented. An interesting approach of using a smartphone to obtain reliable bathymetric data for 3D modelling is given in [10], as another example. This shows that the use of SBESs can significantly reduce costs while maintaining the required data accuracy in some cases and that SBESs can still be a topic for research.

MBES systems have become widely used due to their numerous assets, including wide coverage and high accuracy (especially with technology development). However, as complex systems, they have disadvantages compared to simpler ones. For example, they require additional supporting sensors, and they are more vulnerable to environmental factors. Data processing in MBESs is also more complicated and requires specialized knowledge, including the important issue of data filtration. This basically means eliminating false signals and erroneous soundings based on certain assumptions to provide reliable information [11]. Each hydrographic dataset must be postprocessed with some kind of data filtration prior to the visualization step. The typical flow for this postprocessing stage involves the correction of raw data with basic settings and environmental factors (offsets, water level, etc.), filtration with the use of specially designed filters to reject unreliable and false data, and validation of the results, usually by an experienced hydrographer. The filtration step is the most time-consuming and complicated part of the process. Many authors refer to this step as data cleaning [5,12,13]. Data filtering was traditionally carried out manually by an experienced hydrographer; however, as technology and computer capabilities have evolved, more and more automatic approaches have been developed. The authors of [5] found that more than 30 different algorithms have been published for this purpose, with the aim of reducing the human workload required for manual processing. 

The basic goals of MBES data filtering in modern hydrographic systems and software are increasing data quality and data reduction [2]. MBESs provide dense point clouds, which can be treated as big data. Therefore, proper techniques for data reduction have been proposed in some approaches [14,15]. However, in most publications, the main impact of filtering has been on data cleaning, and the algorithms have been focused on data quality. Data cleaning mostly refers to outlier removal in the case of MBES-related research, and the criterion for defining outliers can be different in various approaches. Ref. [12] stated that “an outlier is any point *z* exceeding a given tolerance (not necessarily constant) from the true ocean bottom at the same location that z is measured, otherwise z is said to be valid”. A wider definition was given 20 years later in [5], where outliers were defined as “an unordinary value, an unusual measure, an exception”. This shows an evolution in the proposed approaches in the scientific community for MBES data cleaning, from relatively simple point depth analysis to advanced algorithms based on statistical, spatial, temporal, or surface analysis. In general, ref. [5] categorized data-cleaning approaches based on outliers into two categories: data-oriented and surface-oriented approaches. In the first group, the focus is on the manipulation of the data themselves and finding patterns which can be used to find outliers. These are in most cases statistically driven; however, other approaches, such as distance-based, density-based, and clustering-based approaches, can also be used. The second group relies on a surface model to which the soundings are compared for potential removal. Most publications include methods from one of these groups.

Data-oriented approaches are represented in many applications; for example, ref. [12] proposes outlier removal based on various statistics, such as minimum and maximum values, as well as covariance filters. Additionally, a screening filter and local matrix validation were proposed. The need for filtration was underlined, especially for shallow waters. In [13], as another example of a median filter approach, which is a modification of typical image filtering, it was proven that it can be useful in reducing random noise while preserving edges in an image. An interesting and popular approach to data-oriented cleaning is the CUBE algorithm, proposed in [16]. It has been implemented in several hydrographic software applications. The method attributes each sounding with an estimate of vertical and horizontal error and then uses a model of information propagation to transfer information about the depth from each sounding to its local neighborhood. A relatively new alternative is the Rolling Circle Transform algorithm proposed by [17], which takes into account the statistical characteristics of the bathymetric data in Ping and analyzes the concave and convex properties of multibeam bathymetric data.

The most popular surface-oriented algorithms are those provided in [18,19]. In [18], an approximation of bathymetry surface is searched that best explains the observed sonar data given statistical models for acoustic noise, backscatter, spatial correlation of the seafloor and prior models for seafloor shape. A search procedure recursively locates the most probable surface by maximizing a surface-scoring function that quantifies these factors. In [19], using a triangulated map of soundings and noise and then a coarse-to-fine algorithm, outliers were detected. Thus, the continuous approximating surface was iteratively refined. In [20], an interesting filter was proposed based on geostatistical relationships, instead of only statistical relationships. Three algorithms were proposed: two analyzing the local distribution of depths and one looking globally at Kriging cross-validation. The thin-plate spline proposal was given in [21] and will be further elaborated in this research. A complex method for data cleaning was also proposed in [11] that combines filtering of the optimal reference curved surface and the trend surface.

The authors of [5] emphasized that even for methods that have been largely accepted by the hydrographic community, it appears that none of the techniques are better than the others, though they may be better adapted to their own native conditions of use. All of these algorithms were validated in their papers. Usually, however, one paper provides an analysis of one method for a particular dataset, focusing on its own approach. Sometimes, a comparison with other selected methods is mad. The noticeable gap in these references, mentioned also in [5], is that comparative analyses are limited, partly as a result of a lack of synthetic and real data benchmarks.

The evaluation of data filtering quality is also an interesting scientific problem. As reviewed in [5], the basic validation techniques are visual inspection of DBMs, comparison of soundings after filtration with manual expert processing (usually based on statistics), and comparison with a reference survey, usually made with more accurate devices providing a kind of ground truth. For example, in [11], statistics for points comparing raw and manually filtered data were given. In [12], depth values for points were assessed by experts as being good, bad, or unknown. In [13], a comparison of surfaces filtered manually and automatically was made. In [20], the rate of detection and the rate of extraction were calculated. In [16], visual inspection followed by point-wise differences in depth was proposed. A similar approach was proposed in [17] but was supported with RMSE calculations. Statistical comparisons with a reference surface were also used in [22]. It can be noted, therefore, that evaluation basically relies on comparison with manually filtered data, and usually one kind of evaluation is given in a paper. It is not clear what the advantages and disadvantages of these particular approaches may be in given use-cases.

With these gaps in mind, the aim of the study was to analyze bathymetric data filtration quality for various filter parameters in order to automatically remove spikes in difficult areas as accurately as possible. We had two goals in conducting this research. First, we provide a comparative analysis of various filtering methods as parts of different processing groups—two surface-oriented and one data-oriented—on one benchmark surface. We chose to perform the research on data for a difficult and diverse harbor area which would be a challenge for filtering methods. Then, we evaluated them in an advanced manner, utilizing four different approaches to data evaluation. Alongside the traditionally used methods, one method based on cross-sections was proposed. Thus, a complex approach to evaluating data filtering has been proposed. We first assessed the filtering quality of the analyzed methods, aiming to find correlations and relationships between the evaluation and filtering methods to achieve an evaluation method assessment. QPS Qimera 2.4.4 software was used as a research tool for the implementation of the analysis.

The rest of the paper is organized as follows: in Section 2, the scope of filtration is briefly given, then the methods selected for the research are described and the methodology of the quality assessment is presented, followed by the research methodology. In Section 3, the results of the research are given, including tables, figures, and the assessment itself, with the proposed evaluation methods. Section 4 covers a discussion of the results, and the conclusions are drawn in Section 5. 

## 2. Materials and Methods

In this section, we focus on three selected filtration methods and four selected assessment methods. The overall research methodology is given in Section 2.4.

### 2.1. The Scope of Multibeam Bathymetric Data Filtration

Bathymetric data are among the most commonly collected hydrographic data. According to the formal definition of the International Association of Oil & Gas Producers (OGP), bathymetric data represent the distance measured perpendicular to a reference surface [23].

The operation of a multibeam echosounder is based on the generation of multiple hydroacoustic beams, creating a “swath” on the surface of the bottom. The size of the “swath” can be equal to 2 to 12 times the water depth. The advantages of using a multibeam echosounder include the abovementioned possibility of 100% data coverage of the bottom as well as the relatively short amount of time required for surveying, which significantly improves the ability to survey large areas of the seabed in a relatively short period of time. When using a multibeam echosounder in hydrographic surveys, it is also necessary to keep in mind the factors affecting the total depth measurement error, which include the speed of sound in the water, which depends on the temperature, depth, and salinity of the water area; the time of measurement; the slope of the bottom; the movement of the vessel; tides; and the depth of the draft of the echosounder transducer.

Taking all these factors into account, it is crucial that each collected bathymetric datapoint is submitted to a postprocessing stage, meaning that additional correction and filtration of data is performed after the acquisition of the raw data. Postprocessing of bathymetric data is usually implemented in the following steps:

- STEP 1: The correction of survey data by basic elements affecting their accuracy. These elements include water levels in the surveyed area (especially for rivers and tidal areas) and the draft of the sonar transducer on the date of measurement. During the implementation of the first step of bathymetric data postprocessing, it is also necessary to take into account the alignment of the data to the measured profile of the speed of sound in the water, the offsets of the other measuring devices entered, and the correction of the positioning of the vessel.
- STEP 2: The hydrographer performs the filtration using filters designed for data postprocessing. These include filtration methods that work by removing incorrect depth indications; for example, Surface Spline filtration, Reject Outliers 1D, and Reject Outliers 3D. While selecting the filters each time, it should be kept in mind that they must be selected individually for the surveyed area with reference to the measurement conditions and the expected results.
- STEP 3: An experienced hydrographic surveyor reviews the data and validates the results [24].

It should be highlighted that the essence of bathymetric data filtration is the elimination of false data, which have been classified as measurement errors obtained for the bottom surface. Bathymetric data from multibeam echosounders especially require the application of filtration due to the number of incorrect data created by interference in the hydroacoustic channel.

Current software for collecting and processing bathymetric data offers a wide range of semi-automatic filters which are based on built-in algorithms, with the possibility of manual choice of parameters. The procedure for filtering bathymetric data from survey data with incorrectly recorded depths, which are not sufficient to ensure the safety of navigation, makes it possible to obtain data that reliably reflect the seabed of the surveyed area. Although numerous filters are implemented in software, it should be noted that the experience of the hydrographer is still crucial for proper data handling, especially as, until now, few deep-learning AI approaches to MBES data cleaning have been published [5].

### 2.2. Selected Filtration Methods

Filtration of bathymetric data can be conducted using several software applications which implement filtering algorithms. The most commonly used filtering methods allow the following:

- Acceptance or rejection of additional depth points in a selected area, which include those points that the echosounder has recorded below and above the number provided by the bottom-detection algorithms;
- Acceptance or rejection of the user’s depth points in the selected area, which include those points generated by the user in the water-column view;
- Rejection of depth points using a triangulated irregular network (TIN), leaving only the points that are necessary to determine the shape of the bottom surface;
- Rejection of depth points that are below and above the set depth;
- Rejection of depth points that are located further away than a specified vertical distance from the average depth of the regular rectangular DBM (usually called GRID);
- Rejection of depth points for which the hydroacoustic beam is outside the angular range set by the user;
- Rejection of all depth points with an intensity outside the specified range.

#### 2.2.1. Reject Outliers

One of the simplest, yet most popular and in many cases effective, approaches is just to remove the points which are too far from the generally understood surface. In the software used in this study (QPS Qimera), the implementation of this method is called *Reject Outliers (1D),* and it basically works by rejecting, based on a user-specified threshold, those depth points that are further from the average depth in the GRID mesh. This was the first filtration method used in this research. The following parameters were available for this method of filtration:

- Vertical Threshold: All soundings greater than the parameter are rejected;
- Grid Resolution: Based on the parameters, the grid is created on-the-fly;
- Minimum Points Per Grid Cell;
- Dynamically Update Grid-Cell Depths: After rejecting the most extreme soundings, the cell’s mean depth is updated.

With this in mind and based on the systematic approach given in [5], this is an example of a data-oriented approach. This particular implementation integrates a statistically based approach with density-based evaluation in its algorithm. Nevertheless, this method is representative of the data-oriented concept of data filtration used in our research.

#### 2.2.2. Decimate to TIN Vertices

The second method used in our research is called *Decimate to TIN Vertices,* and it is an example of a surface-oriented approach in which an algorithm removes points which do not follow the expected pattern. This filter removes depth points using an irregular triangle network, leaving as many points as necessary to build the surface. In the first step, a TIN model is created and is then subdivided by inserting outlier depth points from the surface. This process is repeated until there are no depth points that are farther than the set tolerance value of the TIN network. In the second stage, all depth points that do not form a TIN model are rejected. Thus, the method is similar to the popular approach proposed in [17]. The following parameters are available for this method of filtration:

- Absolute Tolerance;
- Tolerance as % of Depth: To disable the relative tolerance, the user should set this parameter to zero;
- Maximum Link Distance: The impact on the density of the TIN.

In our research, this method represents a surface-oriented method based on local analysis of the neighborhood.

#### 2.2.3. Thin-Plate Spline

The last method examined in this research is based on the *thin-plate spline* algorithm. The basic idea of it is to try to fit the created surface to the data provided by the multibeam echosounder and then remove those depth points that are located too far from it. Thin-plate spline is a basis function for representing coordinate transformations. It is also the 2D generalization of the cubic version of spline. The drawbacks of this algorithm include that its implementation requires the inversion of a large, dense matrix of size p × p, where p is the number of points in the dataset. There are a few methods that have been developed to solve this problem with connections to related approaches in the area of Gaussian RBF networks. An elaboration on this issue can be found in [21]. The basic principle of the thin-plate spline method is based on the equations listed below, in which the height value is calculated:

*z(x,y) = −U(r) = −r*^2^*log r*^2^ (1)

where *r* is the distance $\sqrt{x^{2}+y^{2}}\;\mathrm{from}\;\mathrm{the}$ Cartesian origin. The maximum value of the surface is achieved along a circle of radius 1.

(2) $$\Delta^{2}U={\left(\frac{\partial^{2}}{\partial x^{2}}+\frac{\partial^{2}}{\partial y^{2}}\right)}^{2}U\;\propto \delta_{\left(0,0\right)}$$

This basis function is the natural generalization to two dimensions of the function |*x*|^3^ that underlies the familiar one-dimensional cubic spline [25]. This method is an example of global surface-oriented filtering.

In the practical approach of spline methods, data filtration is implemented in two steps:

- STEP 1: At this step of data filtering, it is important to identify large errors (spikes) because the generated surface will be deformed if large deviations exist. The abovementioned elements have to be removed to obtain a spline that will represent the actual bottom surface. A surface spline is considered matched if the mean square value of all differences between the surface and the real points is less than a fixed threshold, which is the first parameter of the filter and is expressed as a certain percentage of the depth. This value is often called the deviation in practical implementations (e.g., in QPS software). The threshold is the first parameter of the filter and is expressed as a percentage of the depth.

If the deviation is higher than the set threshold, then the following elements of the dataset are removed during surface calculation:

○ The point with the maximum depth;
○ The point with the minimum depth;
○ The point with the largest positive difference from the surface;
○ The point with the largest negative difference from the surface.

Then, the surface area is recalculated. If the deviation is below the threshold, the surface is defined as matched. Otherwise, the process of removing the abovementioned points is repeated. This process is repeated until the deviation target is reached or more than half of the raw data points have been discarded. If a matched surface is available, all points that have a greater difference than the set point are filtered out. This parameter is the SD coefficient of the first step.

- STEP 2: In this step, all remaining points are used to create a new spline surface. The second step is used to clear all multibeam echosounder indications that are located further than the calculated distance from the new spline surface. The parameter used in this case was the SD coefficient of the second step.

In this research, we investigated one of the practical implementations of the thin-plate spline algorithm, which is called *QPS Surface Spline* in the QPS software used. The type of spline was chosen from a list of presets, which are related to the IHO S-44 standard. The 3D spline surface is a representation of the local topography built using approximately 50–100 soundings at a time. The surface is calculated using a weighted least squares method with the available soundings. The criteria for spike detection depend on the expected sounding vertical accuracy, which scales with filter strength. The following parameters are available for this filtration method:

- Spline Type: This represents the strength of the algorithm: strong filters filter more data than weak filters;
- ROV Depth: This is the positive downward, which is added to the sounding depth to reference the sounding relative to the sensor;
- Reference Depth: This can be used to adjust the reduced soundings to obtain values relative to the water level;
- Rejection Preference: This is used to specify which soundings are rejected in relation to the computed spline depth.

#### 2.2.4. Method Variants in the Research

The goal of the method selection for the processing was to examine various approaches to data filtration. The preference was to choose methods implemented within one software program to avoid any additional problems, inaccuracies, or inconsistencies related to internal preprocessing, calibration, and standardization within various software environments. These methods were selected as representative examples.

To meet the needs of the research, variants of three selected methods were proposed. In QPS Surface Spline, the variants were based on the recommendations of the software developer, as presented in Table 1. These variants used different types of spline, which are normally used for different areas.

In the Decimate to TIN Vertices method, the variants were based on various parameters, such as absolute tolerance, tolerance given as percentage of depth, and maximum link distance. The values taken in the variants are given in Table 2. 

In the Reject Outliers (1D) method, the basic parameters were vertical threshold, grid resolution, and minimum points per grid. The variants were defined as in Table 3. In all cases, dynamic updating of the grid cells was chosen.

This selection of variants allowed us to analyze the influence of the various methods as well as the influence of their various parameters.

### 2.3. Methodology of Quality Assesment

The quality of bathymetric data filtering is crucial for further processing towards digital bottom model calculation and for final products, such as electronic nautical charts (ENCs) or bathymetric plots [26]. Perfectly filtered data should not include false detections; however, bottom diversity should be represented with the best possible accuracy. Filtration is a process in which a compromise in the setting of the parameters is needed. Strict values result in the deletion of all false echoes; however, proper data representing small bottom variations can also be deleted with such settings. Therefore, the selection of optimal filtration parameters is usually made empirically, based on the hydrographer’s knowledge. This kind of knowledge is not yet present in automatic filtration methods, which basically just follow numerical algorithms. It is therefore necessary to decide how to evaluate filtered data and how to assess the quality of bathymetric data filtering. 

The most common way to achieve this is to compare data to some kind of reference surface or data, usually based on a digital bottom model, but often also on a direct analysis of the point cloud which has not been interpolated. In many cases, the reference is set following manual filtration by an experienced hydrographer. In other approaches, visual assessment by a hydrographer is a benchmark for quality assessment.

In this research, we carried out comprehensive quality assessment using four different assessment methods. This allowed us both to assess the data and compare the evaluation methods with each other. The methods used were as follows:

- Differential surface statistical analysis;
- Visual assessment of surface roughness and artefacts;
- Comparison with reference control points;
- Selected cross-section analysis.

A differential surface was calculated by comparing the achieved DBM with the reference surface, which was elaborated based on an expert’s manual filtration. Typical statistics were calculated for different surfaces, including the means and standard deviations. Additionally, the number of points that were different from 0.00 m and the extent of the three sigma boundaries were analyzed. The final step in the data analysis was to visually analyze the selected differential surfaces and assess the amount and placement of interpolation errors. This allowed the analysis of global differentiation of calculated DBMs from the reference. Thus, an assessment of filtration related to the entire surface could be made. 

Visual analysis again took into account the entire surface; however, the goal was also to identify local artefacts based on an expert’s knowledge and experience. Visual assessment of the obtained numerical terrain model allowed preliminary conclusions to be drawn about the bottom course in the surveyed area. According to the criteria adopted for the analysis, it gave more or less accurate results. The basic task here was to analyze general roughness or smoothness of the surface. Additionally, data were analyzed by taking into account the visual aspect of the created DBM and the number and placement of the removed data for the surface. The results of the analysis of the filtering methods in each test field were then compiled. Those filtering methods that obtained the best results were assigned a value of 1, and the others were assigned a value of 0. This made it possible to determine which DBM most accurately represented reality.

The method of analysis using reference point maps consisted in the creation of maps where Z values were generated for points with specific XY coordinates. By using the above procedure, it was possible to compare data using the third coordinate reading. The method shows the local similarity of models after filtration to reference data. A crucial issue regarding the usefulness of this method is the proper selection of test control points. These should be placed in characteristic places on the surface, such as slopes, holes, or small objects. Using a qualitative indicator of control points as the first step, it was decided to determine ten locations with known coordinates. Then, the depth at each of the determined locations was read for the surfaces created using the chosen filtration methods. This procedure was also repeated on the surface chosen as the reference. The final step was to calculate the difference between the reference depth and the resulting depth after filtration. 

Cross-sections of the area were used to accurately analyze the studied problem based on routing the cross-section profiles through each of the created surfaces and the reference surface and then analyzing the received data in two steps. When using this method of data analysis, it should be noted that it allows accurate data analysis locally but tends to show trends in desired user-decided directions. Therefore, it can be beneficial for both local point methods and global surface methods, as it can highlight additional trends. Step one involves visual analysis for each dataset and then the identification of the one filtering method from each set that most closely represents the bottom. Step two consists of choosing the criteria for comparison, such as the number of places differing from the reference surface by a certain value. Then, it is necessary to assign a score to each criterion, using which it is possible to select a filtration method to reflect the bottom course as precisely as possible.

All four approaches of assessment were adopted in this paper to provide a complex comprehensive analysis and to determine the benefits of each of them. 

### 2.4. Research Methodology

**Figure 1:**
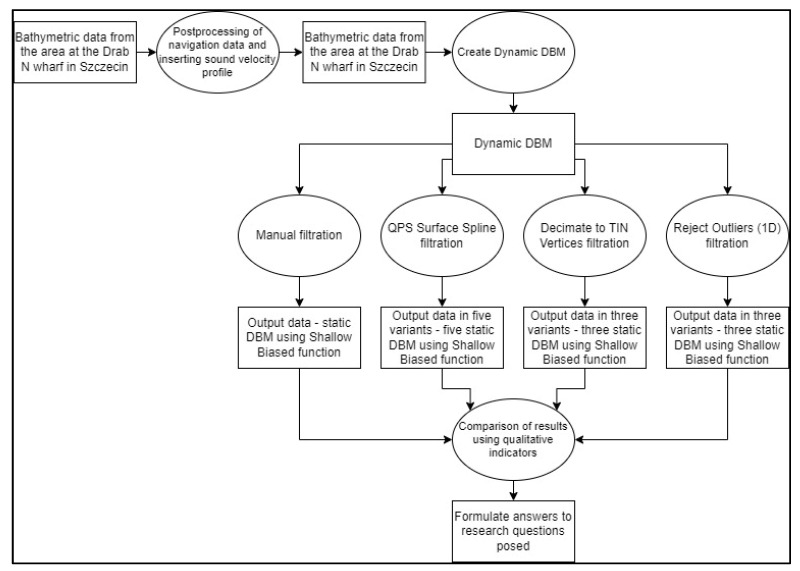
Data-flow diagram.

The general aim of the study was achieved by analyzing bathymetric data filtration quality for various filter parameters. Simultaneously, various quality-assessment methods were used. The diagram in Figure 1 shows the data flow scheme that was adopted for the research. Finally, after finalizing the data flow, quality assessment was performed with the use of the formerly presented indicators.

For filtration quality assessment, it was decided to formulate three research questions:

- How does the filtering parameter selection of bathymetric data affect the DTM built on their basis?
- Do default filtration parameters recommended by software developers for a given class of survey areas meet the requirements for the quality DTM set by the IHO-S44 standard?
- How should filtration parameters be selected for areas that it is difficult to apply automatic data postprocessing to, e.g., along wharves or isolated elements protruding above the bottom?

To obtain answers to the research questions listed above, it was decided to choose a survey area characterized by a high degree of difficulty during the postprocessing of bathymetric data. Difficulties in postprocessing occur, for instance, in water areas that:

- Do not have a flat bottom;
- Are located next to a wharf or other hydrotechnical construction;
- Have been dredged shortly before the bathymetric surveys, resulting in more scattered survey data;
- Have shallow depths;
- Have an irregular shoreline covered with vegetation.

**Figure 2:**
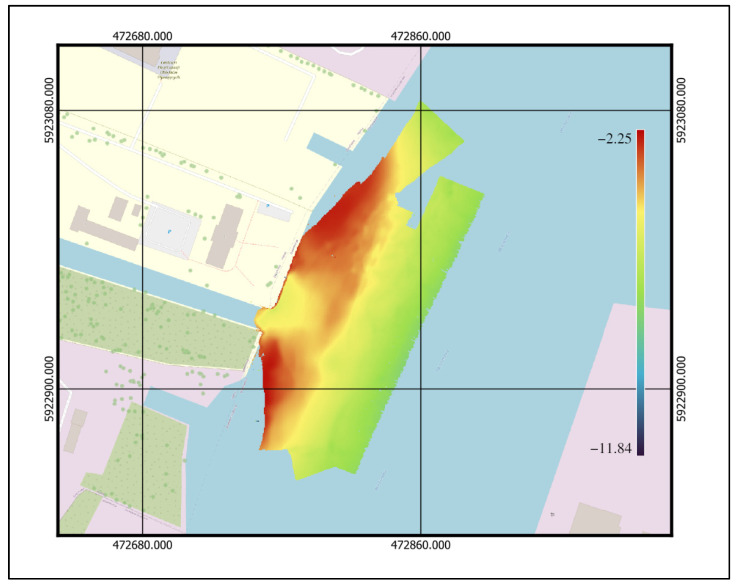
The survey area in Szczecin (UTM coordinates given).

The chosen data that were analyzed met most of the abovementioned criteria, i.e., they were obtained from bathymetric measurements made near the hydrotechnical construction of the Drab N wharf located on the West Oder River in Szczecin. The area was characterized by an irregularly shaped bottom, and it was shallow, but not flat, with a depth mainly between two and seven meters. The area is presented in Figure 2.

The data used for the tests were acquired using the R2Sonic 2022 MBES. Positioning during bathymetric surveys was performed using GNSS technology with RTK (real-time kinematic) corrections provided by Trimble VRS (Virtual Reference Station). The accuracy of the position was determined to be 0.03 m (horizontal) and 0.05 m (vertical). The measurements referred to the average water level established at the Szczecin Long Bridge water gauge, which was the closest one to the survey area. The mean reference water level was established with long-term GNSS RTK observations. Reading of the water level was performed online by an automatic sensor and was provided with an official online service by the Polish Institute of Meteorology and Water Management. Based on this information, the errors for the water level were expected to be less than 0.01 m. 

**Figure 3:**
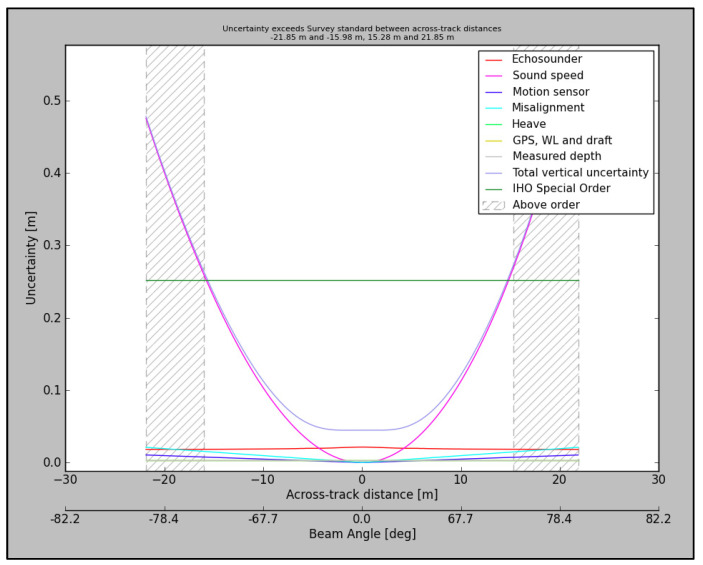
Total vertical uncertainty for the survey in the research.

Survey error is a very important aspect that affects the proper analysis of bathymetric surveys but also putting the results of the research in proper perspective. IHO in [8] has defined several survey orders considering accuracy. In the case of areas where under-keel clearance is critical, a special order is recommended. It assumes that total vertical uncertainty (TVU) at a 95% confidence level is not higher than 0.25 m + factor related to depth, which is small in shallow areas. For example, for a depth of 4 m the allowed TVU is 0.252 m, and for a depth of 10 m the allowed TVU is 0.261. With this in mind, we estimated the TVU for our hardware and the environmental configuration using the AMUST 3.0.0 software tool produced by TU Delft. This is a software tool dedicated to the a priori modelling of MBES survey uncertainty. The results are presented graphically in Figure 3. The model includes the types of sensors used and other corrections (including water-level error).

It can be noticed that the basic measurement error has a value of 0.456 m and that the uncertainty increases with the range from the sensor. However, bearing in mind the dense survey profiles and thus the relatively small effective swath range in our case, this value can be used as an accuracy indicator in further analysis. Finally, it was decided to use two quality-assessment thresholds: 0.45 m, representing a priori TVU, and 0.252 m, representing IHO requirements for a special-order survey.

For the postprocessing, QPS Qimera software was used, applying the variants of filtration methods described in Section 2.2.2.

**Figure 4:**
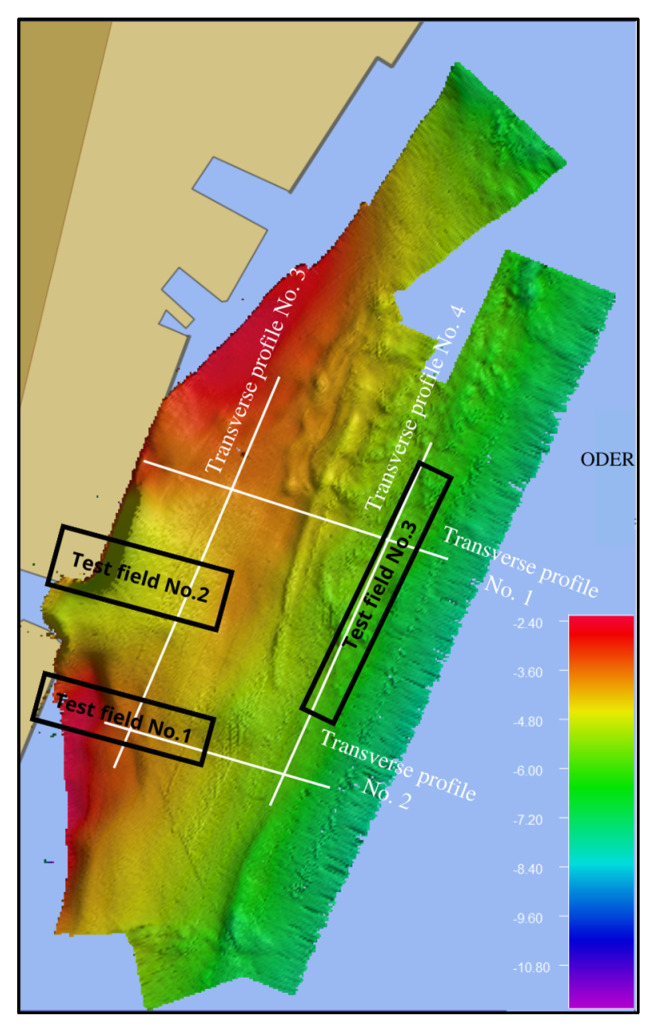
Test fields.

The methods were assessed using the following quality indicators:

- The difference between the area generated from the filtered data and the area generated from the manually cleaned data, taken as reference basic statistics also related to TVU thresholds;
- Expert visual assessment of the test fields: It was decided to create three test fields, located in different parts of the surveyed area, so that it would be possible to test the filtering effects of individual filtration methods in as many cases as possible. The test fields are presented in Figure 4. The characteristics of the bottoms in the areas covered by the test fields are as follows:
  ○ Test field no. 1: Visible measurement errors caused by the set range of the MBES and caused by the slope, which is an important element of the bottom;
  ○ Test field no. 2: This area includes two important elements of the surveyed area, i.e., a large trim (a drop-off point for vessels) and a highly visible wharf wall;
  ○ Test field no. 3: This area is flat with spikes protruding above the bottom.
- Depth differences at control points;
- Transverse profiles (cross-sections): Tracing a line along a specific area, these allowed comparison of the different bottom profiles and depths, although for a small area.

The reference for the quality-indicator assessments was a digital bottom model created based on manual filtration by an expert (an experienced A-category-certified hydrographer).

To create the digital bottom model, it was decided to use QIMERA 2.4.4 software. The software allows users to create a surface with a GRID model, using the following methods:

- Weighted Moving Average: This is the default weighted-average algorithm;
- Shallow Biased: The smallest value in a GRID cell is used;
- Median Filtered: The average value in a GRID cell is used;
- Deep: The deepest value in the GRID cell is used.

Then, a preliminary analysis of the GRID mesh-creation methods was conducted, which was based on a visual analysis of the surfaces created using all methods. This visual assessment included verification of the number and distribution of spikes on the DBM and also verification of whether the bathymetric data on the wharf had been removed. After this preliminary analysis, it was decided to choose the “Shallow Biased” method, and a value of 0.25 m was set as the mesh size of the GRID mesh so that as many bottom features as possible that could be a potential threat to navigational safety could be indicated on the generated model.

Although the main goal of the study was related to filtration quality, it was also decided to analyze the first processing time for the different filtration methods, which is one of the key implementation issues.

## 3. Results

The processing time for the analyzed filtration methods depended primarily on two factors: the number of data and the characteristics of the computer used. In this case, the point cloud consisted of 284,008 depth points, and the computer used was an HP Z4 workstation. Time measurements are presented in Table 4.

Processing times varied from 5 to nearly 8 min, which are both relatively short periods for off-line processing. However, it should be noted that spline methods are noticeably faster than the others. 

### 3.1. Differential Surface Analysis

Four quality indicators were used to analyze the data, as presented in the research methodology. For the first, eleven differential surfaces were calculated—one for each variant—and the basic statistics were computed. These are presented in Table 5. The total number of depth points in the reference surface was 282,151, and the depths are represented with negative values in the table. It can be noticed that the lowest values in some cases are about 14 m, which is unlikely for the study area. These values are examples of so called “spikes”, which were not automatically filtered by the data-cleaning method.

Table 5 includes even the smallest deviations from the reference surface; however, it should be noted that in practice mean errors of 20 cm or more are not uncommon for bathymetric surveys. Therefore, for deeper understanding of the filtration quality and to put the results in appropriate perspective, additional indicators were proposed, as described in the research methodology. In Table 6, the values calculated for a threshold related to the estimated TVU (called the TVU threshold) and for a threshold related to the IHO Special Order (called the IHO SO threshold) are presented. The thresholds were applied to the different surfaces.

The results presented in Table 5 and Table 6 show that the selected methods performed generally well and that the number of points exceeding the thresholds was not significant in most cases. However, differences in quality between the methods and their variants can be observed. Based on these results, the Decimate to TIN Vertices method was excluded from further analyses. The criterion was the 3-sigma rule, and the chosen thresholds were 0.120 m to 0.150 m. Next, among the other filtering methods, three were singled out that had the smallest number of points that differed from 0.00 m and the smallest number of points exceeding the TVU threshold. These included Weak Spline and Reject Outliers (1D)—variants 1 and 2. The latter method performed significantly better than the others, taking into account the lowest and highest values for differential surfaces.

**Figure 5:**
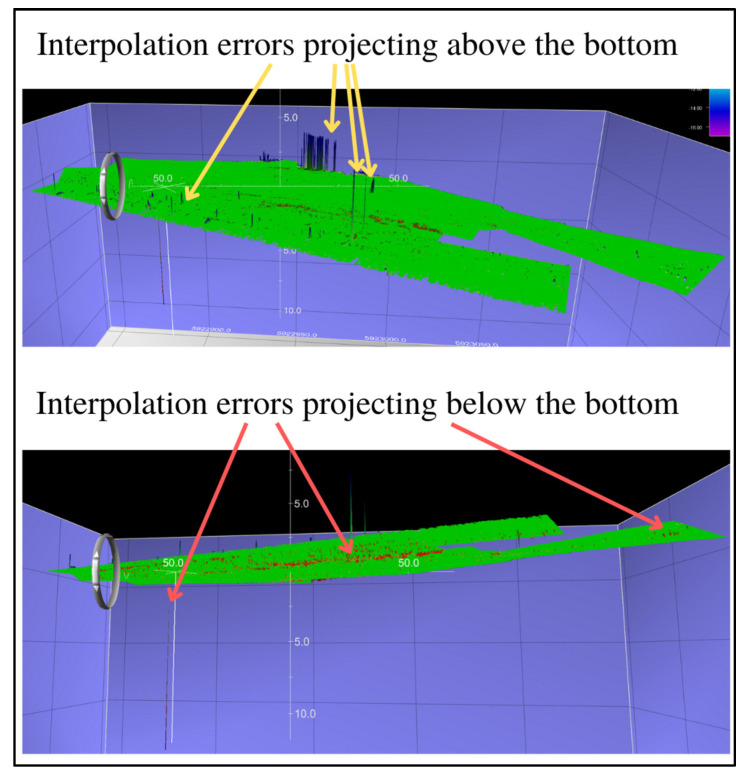
The surface created by the comparison of the reference surface and the surface created by the Weak Spline filter.

**Figure 6:**
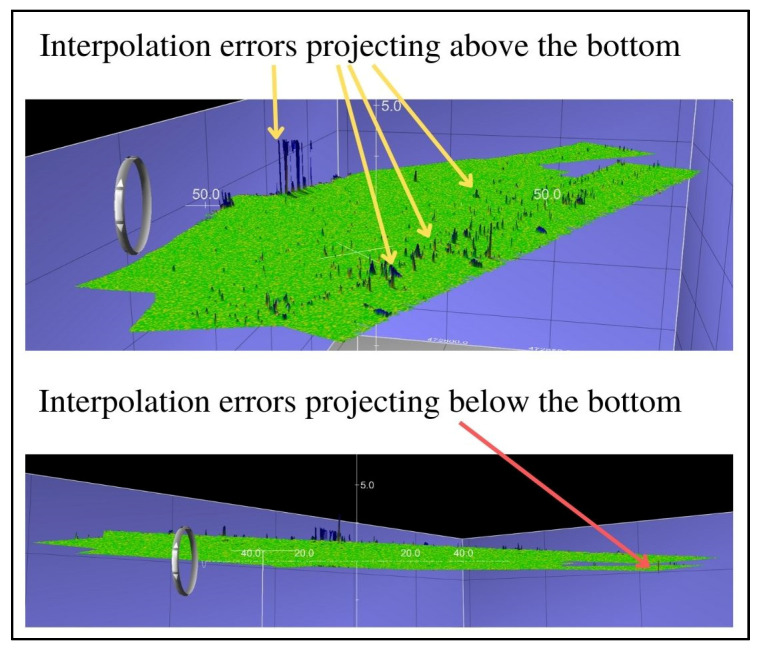
The surface created by the comparison of the reference surface and the surface created by the Reject Outliers—variant 1 filter.

**Figure 7:**
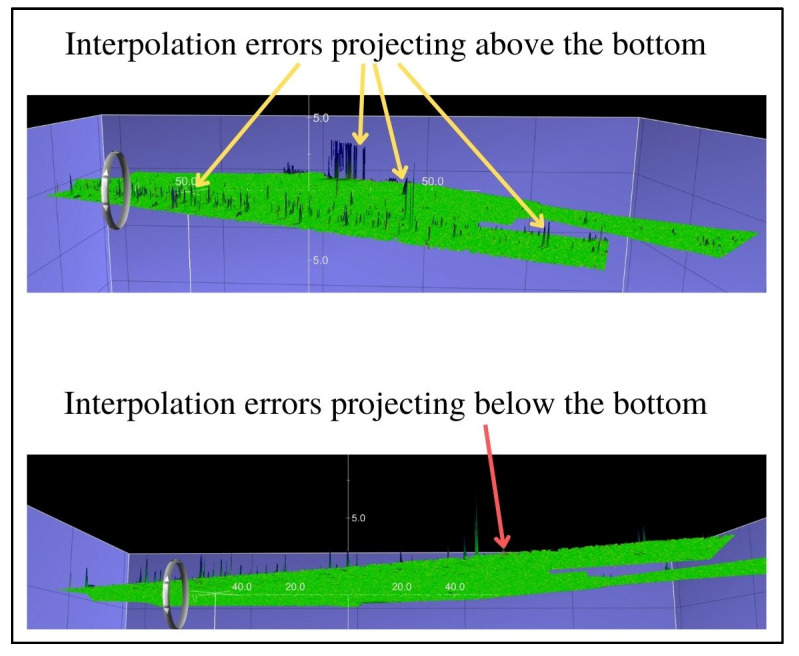
The surface created by the comparison of the reference surface and the surface created by the Reject Outliers—variant 2 filter.

The final step in this method of verification of the filtering methods was additional visual analysis of the selected surfaces, during which it was noted that all surfaces had small remaining interpolation errors projecting above the bottom, while the surface created with the Weak Spline filter also left small interpolation errors projecting below the bottom. Visualizations of the interpolation errors for the abovementioned differential surfaces are given in Figure 5, Figure 6 and Figure 7.

### 3.2. Expert Visual Assesment of Test Fields

The second method for quality assessment was expert visual evaluation of the data for the selected test fields. After seeing the filtering results, the following data analysis criteria were adopted:

- Visual evaluation of the created DBM;
- Visual evaluation of the number of data deleted from the DBM;
- Visual evaluation of the distribution of data deleted from the DBM.

**Figure 8:**
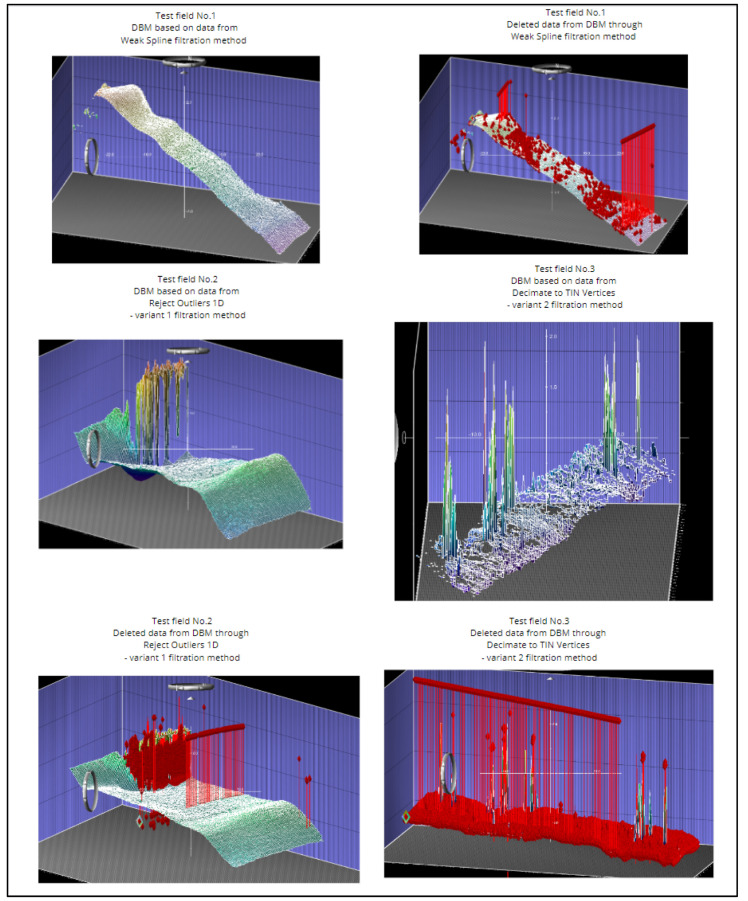
DBMs in test fields as given in QPS Qimera software. Red color is showing deleted data.

Below, we present selected examples of DBMs in each test field and examples of visualization of the data removed in the sample filtering methods (Figure 8).

After analyzing the data with the analysis criteria in mind, it was decided to summarize the results in two steps. 

In the first step, the filtering results were divided individually for the test fields, and the following findings are presented:

- In test field no. 1, the best results were obtained using the Very Weak Spline and Weak Spline filtering methods.
- In test field no. 2, the best results were obtained using the Reject Outliers (1D)—variant 1 filtering method.
- In test field no. 3, the best results were obtained using the Very Weak Spline and Reject Outliers (1D)—variant 1 filtering methods.

The abovementioned results were obtained by analyzing each of the filtration methods, from which the following was concluded:

- The Very Weak Spline and Weak Spline filtration methods were characterized by a small number of removed data in test field no. 1, which were distributed evenly, and the DBM correctly represented the shape of the bottom.
- Filtration by the Reject Outliers 1D method—variant 1 was characterized by a small number of removed data in test field no. 2, which were located mainly at the wharf as expected, and the DBM correctly represented the shape of the bottom.
- Only Very Weak Spline and Reject Outliers filtrations in test field no. 3 were characterized by a small number of data removed from the DBM, and the removed data were above and below the DBM.

In the second step, a joint analysis of the best methods for the test fields was performed. Taking all the test fields into account, it was found that Very Weak Spline and Reject Outliers (1D)—variant 1 were the best filtration methods according to the test-field visual assessment method.

### 3.3. Assessment Based on Control Points

**Figure 9:**
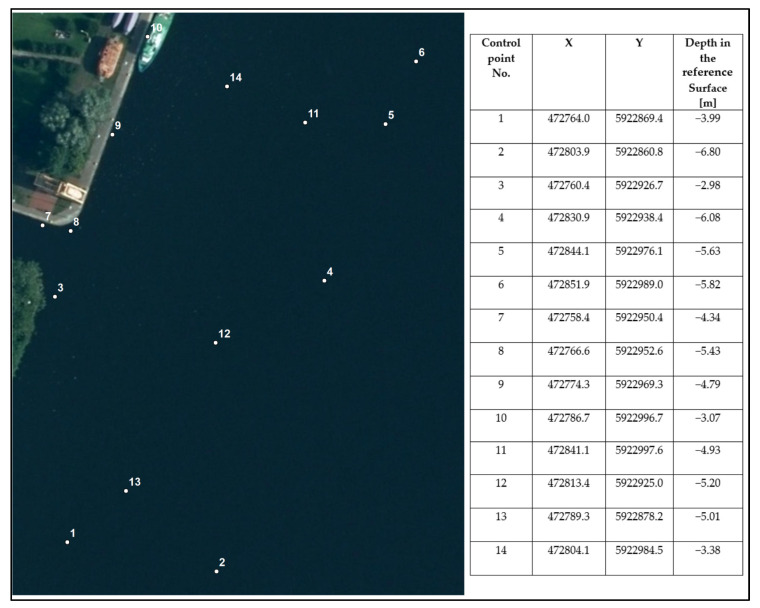
Placement of control points.

The next method used to determine quality was based on the control points of a specified longitude and latitude. The depth values of these points were compared. The control points were selected to represent various parts of the survey area: close to the berth, in the shallow water with a large gradient, and in the deeper and flatter part. The spatial distribution of the control points is presented in Figure 9.

Table 7 shows the depth differences between the reference surface and the surface after filtration at all control points. 

According to Table 7, the differences in control points were generally small, suggesting that all the methods performed well in this assessment. However, this depends very much on the selection of control points, which should be located in the areas of major interest for the user. Point 14 is an interesting example here, showing that it was possible to find points for which all methods performed poorly. Taking into account the TVU calculations, it can be assumed that such points can be located in the areas which were not double-covered, at the end of swaths. Points 11 and 12 show, on the other hand, that there might be significant differences between the methods themselves. All of these local characteristics have to be considered in the discussion. In the situation analyzed here, the results obtained clearly indicated that the Decimate to TIN Vertices—variant 2 and Decimate to TIN Vertices—variant 3 filtering methods were not able to interpolate values at the points of interest. Reject Outliers showed the best results for most points. Further findings are given in Section 4.

### 3.4. Assessment Based on Cross-Sections

**Figure 10:**
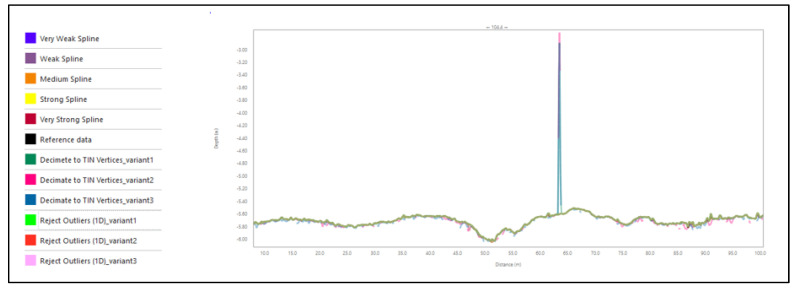
The example cross-sections—part of transverse profile no. 4.

The last method used for quality assessment was data analysis using cross-sections. Example cross-sections are presented in Figure 10. Only part of transverse profile no. 4. is presented to allow the observation of differences; for the entire profile, the scale was too small to visualize them. 

We decided to analyze the data in two steps:

STEP 1: Preliminary visual analysis of the cross-sections in each dataset, followed by selection of one filtering method from each set that most accurately represented the bottom.

STEP 2: A precise numerical analysis of the data, which involved comparing the data selected in step one with the reference data. 

Thus, the criteria adopted for comparison were as follows:

- Criterion 1: Visual analysis;
- Criterion 2: The number of places differing from the reference surface by more than 20 cm in each cross-section;
- Criterion 3: The number of places differing from the reference surface by more than 5 cm in each cross-section.

The results obtained for the cross-sections identified the Reject Outliers (1D) filtering method—variant 1 as the method that produced the DBM most accurately representing reality. The cross-section method requires proper selection of the profiles to be analyzed and in many cases is sensitive to local disturbances. However, a profile can show the performance of a method for various depths and bottom shapes.

## 4. Discussion

The first part of the research showed that the selection of the filtration method and its parameters can have a significant impact on the process’s quality. However, in most cases, good results were obtained. In fact, only the Decimate to TIN Vertices filter performed poorly in some variants. Consideration of the general performance of the methods can allow the selection of the best one in the described quality-assessment stages. A summary is shown in Table 8. 

As can be seen in Table 8, the Reject Outliers (1D)—variant 1 method was one of the best methods in all of the quality assessments. Thus, the conclusion can be formulated that this should be the first choice for filtration. However, in many cases the differences were small, and the specificity of the method and the area should also be considered in the selection. On the other hand, the Reject Outliers methods are also the most time-consuming methods.

Through the analysis carried out in this research, we also reached the following more detailed conclusions:

- The Decimate to TIN Vertices method is more sensitive to parameter changes than the others—the differences between variants were the biggest compared with the other methods.
- In the case of the spline method, the stronger the spline, the bigger were the errors observed.
- Regarding selecting the spline filtering method, built into the QPS software are recommendations of the software manufacturer for a given class of water bodies according to the IHO-S44 standard. These are correct and should be followed, as confirmed in this research. The use of a filter that is too strong gives the opposite of the expected effect, i.e., it does not remove more data but highlights the measurement errors created during the bathymetric survey.
- The Rejects Outliers method in its variants shows similar results, which can be comparable with Weak and Very Weak Spline.
- The Reject Outliers method showed the best performance when analyzing quantitative indicators, while in visual assessments the spline methods performed better in some cases (e.g., test field no. 1).

During this research, an assessment of the evaluation methods themselves was also performed. The *differential surface statistical analysis* method presented a global estimation of data-cleaning efficiency, providing values for the entire surface. This may lead to generalization of the results, but it gives a relatively simple one-value assessment. We decided to support this method with a visual assessment of differential surfaces, focused on the distribution and trends of the interpolation errors, which added new knowledge to the assessment. 

*Visual analysis* of the predicted surfaces was solely used as a second assessment method. Expert assessment of the roughness and usefulness of a surface was directly possible here. This method “naturally” supports global surface-oriented approaches, which tend to smooth surfaces globally. 

The *control points* method, on the other hand, provided information about local errors in selected spots. This selection is crucial for the results, which can be a disadvantage if wrong points are selected. However, it can also be an advantage, as the method is very flexible and can easily be adjusted to user needs. The user can indicate the places or areas at which the prediction is the most important and evaluate the method locally, instead of resorting to or supplementarily employing global assessment methods. Given the above summary, it should be noted that the conclusions about this method should not be generalized, and it is recommended that other methods be used as well in global assessments. This can be recognized from cases in which the number of points did not deviate from the pattern and places for which where there were no data. This may indicate that, compared to the number of points of which the created DBM consisted, the selection of fourteen control points was too low, such that it was not possible to reliably assess the impact of the adopted filtering parameters on the DBM in a global sense with this number of data. However, the differences between the reference surface and the surface after filtration were zero or a few millimeters in most cases, meaning that each of the adopted filtration methods met the set requirements locally, i.e., they are suitable to replace manual filtration. 

The final method we proposed for the evaluation was *cross-section analysis*, including a combination of visual assessment with statistical values. The method is local; however, it tends to present the trends in data and filtration accuracy in selected directions. In many applications, such as river soundings and large gradients, such an analysis can be very useful. This can also provide information about the degradation of information along the beams in swaths. 

It was also noted during the analysis of the assessment methods that both of the methods relying on selected areas, namely, *expert visual assessment for test fields* and *cross-sections,* allowed unambiguous analysis of the area, while the *control points* method focused on the local aspect. Each of these methods can support general statistical evaluation with additional knowledge; however, for global-aspect assessment, it is recommended to use test fields or cross-sections, while, for local verification, properly selected control points would be better. 

Finally, based on our research, there were small trends that global assessment methods favored slightly with surface-oriented filtering, while locally focused methods tended to ally with data-oriented methods. This is an indication of trends, and future research with more methods should be performed to confirm this hypothesis.

## 5. Conclusions

Filtration of bathymetric data is an integral component of hydrographic data postprocessing. Unquestionably, its automation can significantly reduce the time required to obtain cleaned data that can be used to ensure the safety of navigation. Nowadays, hydrographic software developers offer a wide range of types of automatic filtration. However, each water area requires an individual approach when choosing the type of automatic filtration. This should be chosen in relation to the category of water area according to the IHO S-44 standard. Software developers often provide recommendations for methods implemented in their software. However, to achieve the best results in automatic data filtration, it is necessary to adjust the type and parameters of filtration in reference to the studied water area. In special cases, it is also recommended to use a sequence of filters. Therefore, it is important to understand the concepts behind these methods and the quality-assessment possibilities.

This paper aimed to carry out a quality assessment of selected MBES data filtration methods and an evaluation of the quality-assessment process itself as applied to a specific, difficult-to-survey harbor area. Three different filtration methods were chosen and four quality-evaluation indicators were proposed, and performances were analyzed based on real bathymetric data. The research showed that the filtration results depended on the applied methodology. Some of the methods support more local reduction in outliers, while others are more globally oriented. In the case of our research, the best results were achieved with the Reject Outliers (1D)—variant 1 (medium threshold and medium resolution) and Very Weak Spline methods, one being data-oriented, while the other is surface-oriented. 

The findings regarding the evaluation also showed that quality assessment can indicate other aspects of filtration and that various evaluation methods should be used for different purposes. There were no major differences regarding the conclusions of the assessments performed with the various methods; however, some preferences have been noted. The results showed a slight correlation between filtration and evaluation methods. Surface-oriented methods tend to be better in global assessment procedures. This finding, however, is preliminary, as one testing surface is insufficient for the generalization of the findings, and they should be confirmed in further research. However, the present research showed this trend for the specific area studied.

## Figures and Tables

**Table 1:** Use of QPS Surface Spline filtration in relation to IHO S-44.

QPS Surface Spline Filtration Type	Very Weak Spline	Weak Spline	Medium Spline	Strong Spline	Very Strong Spline
Water area category according to IHO S-44	Exclusive order (depth between 0 and 10 m)	Exclusive order (depth between 0 and 20 m)	Special order (depth between 0 and 20 m)	Order 1 (depth between 20 and 50 m)	Order 2 (depth between 20 and 50 m)

**Table 2:** Parameters of the Decimate to TIN Vertices method.

Parameter Name	Variant 1	Variant 2	Variant 3
Absolute Tolerance	0.50 m	0.25 m	0.40 m
Tolerance as % of Depth	50%	0%	30%
Maximum Link Distance	0.10 m	0.50 m	0.30 m

**Table 3:** Parameters of Reject Outliers (1D) method.

Parameter Name	Course 1	Course 2	Course 3
	Variant 1		
Vertical Threshold	6.00 m	2.00 m	0.50 m
Grid Resolution	8.00 m	3.00 m	1.00 m
Minimum Points Per Grid	1	1	1
Dynamically Update Grid-Cell Depths	Yes	Yes	Yes
Variant 2			
Vertical Threshold	12.00 m	5.00 m	1.50 m
Grid Resolution	10.00 m	4.00 m	2.00 m
Minimum Points Per Grid	5	5	5
Dynamically Update Grid-Cell Depths	Yes	Yes	Yes
Variant 3			
Vertical Threshold	4.00 m	3.00 m	1.00 m
Grid Resolution	6.00 m	2.00 m	0.50 m
Minimum Points Per Grid	2	2	2
Dynamically Update Grid-Cell Depths	Yes	Yes	Yes

**Table 4:** Processing times for the different filtration methods.

Filtration Method	Time Required for Data Processing (s)
Very Weak Spline	301
Weak Spline	320
Medium Spline	315
Strong Spline	332
Very Strong Spline	341
Decimate to TIN Vertices—variant 1	426
Decimate to TIN Vertices—variant 2	434
Decimate to TIN Vertices—variant 3	411
Reject Outliers (1D)—variant 1	454
Reject Outliers (1D)—variant 2	421
Reject Outliers (1D)—variant 3	411

**Table 5:** Basic statistics of the differential surfaces.

Name	Highest Value (m)	Lowest Value (m)	Average Value (m)	Number of Points above 0.00 m	Number of Points below 0.00 m	Standard Deviation (3 Sigma Interval) (m)
Very Weak Spline	4.08	−13.82	0.000	1661	4874	0.150 (range from −0.150 to 0.150)
Weak Spline	4.08	−13.82	−0.001	1529	8724	0.150 (range from −0.151 to 0.149)
Medium Spline	4.08	−14.24	0.000	1794	6663	0.180 (range from −0.180 to 0.180)
Strong Spline	3.93	−14.24	−0.001	1662	12,608	0.180 (range from −0.181 to 0.179)
Very Strong Spline	3.96	−14.24	−0001	1807	12,176	0.210 (range from −0.211 to 0.209)
Decimate to TIN Vertices—variant 1	4.46	−14.24	0.003	245	4172	0.270 (range from −0.267 to 0.273)
Decimate to TIN Vertices—variant 2	4.46	−14.24	−0.008	1549	61,632	0.360 (range from −0.368 to 0.352)
Decimate to TIN Vertices—variant 3	4.46	−14.24	−0.008	1552	83,019	0.300 (range from −0.308 to 0.292)
Reject Outliers (1D)—variant 1	2.96	−0.91	0.001	2089	493	0.120 (range from −0.119 to 0.121)
Reject Outliers (1D)—variant 2	3.78	−0.10	0.002	2235	497	0.150 (range from −0.148 to 0.152)
Reject Outliers (1D)—variant 3	2.96	−0.10	0.002	2237	501	0.150 (range from −0.148 to 0.152)

**Table 6:** Statistics for TVU and IHO SO thresholds.

Filtration Method	Number of Depth Points after Filtration	Number of Depth Points above TVU Threshold	Number of Depth Points above IHO SO Threshold	Difference in Number of Points	% of Depth Points above TVU Threshold	% of Depth Points above IHO SO Threshold
Very Weak Spline	282,510	3217	115	359	1.14	0.04
Weak Spline	282,470	4768	101	319	1.69	0.04
Medium Spline	282,476	4033	180	325	1.43	0.06
Strong Spline	282,300	6484	151	149	2.30	0.05
Very Strong Spline	281,589	6555	180	562	2.33	0.06
Decimate to TIN Vertices—variant 1	282,554	1395	391	403	0.49	0.14
Decimate to TIN Vertices—variant 2	159,447	14,801	377	122,704	9.28	0.24
Decimate to TIN Vertices—variant 3	221,017	17,922	200	61,134	8.11	0.09
Reject Outliers (1D)—variant 1	282,509	1249	309	358	0.44	0.11
Reject Outliers (1D)—variant 2	282,504	1327	388	353	0.47	0.14
Reject Outliers (1D)—variant 3	282,546	1323	383	395	0.47	0.14

**Table 7:** Differences between the reference depth and depth after filtration.

	Filtration Method										
	Surface Spline					Decimate to TIN Vertices			Reject Outliers (1D)		
	Very Weak	Weak	Medium	Strong	Very Strong	Var. 1	Var. 2	Var. 3	Var. 1	Var. 2	Var. 3
No.	Difference between Reference Depth and Depth after Filtration (m)										
1	0	0	0	0	0	0	No data	No data	0	0	0
2	0	0	0	0	0	0	0.01	0	0	0	0
3	0	0	0.01	0.02	0	0	No data	0.02	0	0	0
4	0	0	0.01	0	0	0	No data	0.03	0	0	0
5	0	0	0	0	0	0	0.01	0	0	0	0
6	0	0	0	0	0	0	0.01	0	0	0	0
7	0	0	0	0	0	0	0.03	−0.01	0	0	−0.01
8	0	0	0	0	0	0	No data	No data	0	0	0
9	0	0	0	0	0	0	0	0.02	0	0	0
10	0	0	0	0	0	0	No data	No data	0	0	0
11	−0.10	−0.10	−0.11	−0.10	−0.08	0	−0.07	−0.11	0	0	0
12	−0.13	−0.11	0	0	0	0	No data	No data	0	0	0
13	0	0	0	0	0	0	No data	No data	0	0	0
14	0.38	0.38	0.52	0.52	0.52	0.52	0.61	0.51	0.48	0.52	0.52

**Table 8:** Best methods according to various assessment methodologies.

Method of Quality Assastment				
	Differential surface statistical analysis	Visual assessment for surface roughness and artefacts	Comparison with reference control points	Selected cross-section analysis
Filtration methods that achieved the best results	Reject Outliers (1D)—variant 1 Reject Outliers (1D)—variant 2	Very Weak SplineReject Outliers (1D)—variant 1	Decimate to TIN—variant 1 Reject Outliers (1D)—variant 1 Reject Outliers (1D)—variant 2	Reject Outliers (1D)—variant 1

## Data Availability

Not applicable.

## References

[1] Stateczny A., Brebbia C.A., Olivella J. (2000). The neural method of sea bottom shape modelling for the spatial maritime information system. Maritime Engineering and Ports II.

[2] De Wulf A., Constales D., Stal C., Nuttens T. Accuracy Aspects of Processing and Filtering of Multibeam Data: Grid Modeling versus TIN Based Modeling. Proceedings of the FIG Working Week 2012.

[3] Makar A., Specht C., Specht M., Dąbrowski P., Burdziakowski P., Lewicka O. (2020). Seabed Topography Changes in the Sopot Pier Zone in 2010–2018 Influenced by Tombolo Phenomenon. Sensors.

[4] Stateczny A., Bodus-Olkowska I. Sensor Data Fusion Techniques for Environment Modelling. Proceedings of the 16th International Radar Symposium (IRS), International Radar Symposium 2015.

[5] Le Deunf J., Debese N., Schmitt T., Billot R. (2020). A Review of Data Cleaning Approaches in a Hydrographic Framework with a Focus on Bathymetric Multibeam Echosounder Datasets. Geosciences.

[6] Stateczny A., Brebbia C.A., Sciutto G. (2002). Methods of comparative plotting of the ship’s position. Maritime Engineering & Ports III.

[7] Schimel A.C.G., Brown C.J., Ierodiaconou D. (2020). Automated Filtering of Multibeam Water-Column Data to Detect Relative Abundance of Giant Kelp (*Macrocystis pyrifera*). Remote Sens..

[8] International Hydrographic Organization (2020). Standards for Hydrographic Surveys.

[9] Bio A., Conçalves J.A., Magalhães A., Pinheiro J., Bastos L. (2022). Combining Low-Cost Sonar and High-Precision Global Navigation Satellite System for Shallow Water Bathymetry. Estuaries Coasts.

[10] Vozza G., Costantino D., Pepe M., Alfio V.S. (2023). Smart Sensors System Based on Smartphones and Methodology for 3D Modelling in Shallow Water Scenarios. Appl. Syst. Innov..

[11] Yan Y., Yuan L., Ran L., Yin H., Xiao X. Multi-beam Data Automatic Filtering Technology. Proceedings of the 3rd International Conference on Geology, Mapping and Remote Sensing (ICGMRS) 2022.

[12] Lirakis C.B., Bongiovanni K.B. Automated multibeam data cleaning and target detection. Proceedings of the OCEANS 2000 MTS/IEEE Conference and Exhibition. Conference Proceedings (Cat. No.00CH37158).

[13] Mann M., PAgathoklis P., Antoniou A. Automatic outlier detection in multibeam data using median filtering. Proceedings of the IEEE Pacific Rim Conference on Communications, Computers and Signal Processing (IEEE Cat. No.01CH37233).

[14] Wlodarczyk-Sielicka M., Blaszczak-Bak W. (2020). Processing of Bathymetric Data: The Fusion of New Reduction Methods for Spatial Big Data. Sensors.

[15] Wlodarczyk-Sielicka M., Stateczny A., Lubczonek J. (2019). The Reduction Method of Bathymetric Datasets that Preserves True Geodata. Remote Sens..

[16] Calder B.R., Mayer L.A. (2003). Automatic processing of high-rate, high-density multibeam echosounder data. Geochem. Geophys. Geosyst..

[17] Dong J., Peng R., Zhang L., Wang Z. (2016). An Algorithm of Filtering Noises in Multi-beam Data Based on Rolling Circle Transform. Geomat. Inf. Sci. Wuhan Univ..

[18] Arnold J., Shaw S. A surface weaving approach to multibeam depth estimation. Proceedings of the OCEANS’93.

[19] Canepa G., Bergem O., Pace N.G. (2003). A new algorithm for automatic processing of bathymetric data. IEEE J. Ocean. Eng..

[20] Bisquay H., Freulon X., De Fouquet C., Lajaunie C. Multibeam data cleaning for hydrography using geostatistics. Proceedings of the IEEE Oceanic Engineering Society (OCEANS’98).

[21] Donato G., Belongie S., Heyden A., Sparr G., Nielsen M., Johansen P. (2002). Approximate Thin Plate Spline Mappings. Computer Vision—ECCV 2002.

[22] Mohammadloo H.T., Snellen M., Simons D.G. (2020). Assessing the Performance of the Multi-Beam Echo-Sounder Bathymetric Uncertainty Prediction Model. Appl. Sci..

[23] Lekkerkerk H.-J., Haycock T. (2020). Handbook of Offshore Surveying. Volume 3: Acquisition Sensors.

[24] Stateczny A., Rutkowski L., Siekmann J.H., Tadeusiewicz R., Zadeh L.A. (2004). Artificial neural networks for comparative navigation. Artificial Intelligence and Soft Computing–ICAISC 2004.

[25] Bookstein F.L. (1989). Principal warps: Thin-plate splines and the decomposition of deformations. IEEE Trans. Pattern Anal. Mach. Intell..

[26] Stateczny A., Bodus-Olkowska I., Mikulski J. (2014). Hierarchical Hydrographic Data Fusion for Precise Port Electronic Navigational Chart Production. Telematics in the Transport Environment.

